# Rapid screening and identification of novel psychoactive substances using PaperSpray interfaced to high resolution mass spectrometry

**DOI:** 10.1016/j.clinms.2016.08.003

**Published:** 2016-09-28

**Authors:** Joseph Kennedy, Kevin G. Shanks, Kristine Van Natta, Maria C. Prieto Conaway, Justin M. Wiseman, Brian Laughlin, Marta Kozak

**Affiliations:** a Prosolia Inc., Indianapolis, IN, United States; b AIT Laboratories, Indianapolis, IN, United States; c Thermo Fisher Scientific, San Jose, CA, United States

**Keywords:** Synthetic cannabinoids, Novel psychoactive substances, PaperSpray ionization

## Abstract

The simple and rapid detection and identification of designer drugs is of substantial importance to forensic scientists and law enforcement. Although synthetic cathinones, cannabinoids, and other common novel psychoactive substances (NPS) are produced for purposes that do not include human consumption, they are regularly abused. The analysis of these compounds is often achieved using mass spectrometry, but can be complicated by the lack of spectral libraries and the scarcity of simple and reliable sample introduction techniques. PaperSpray® ionization is a new, automated technique for rapid analysis of samples, without chromatography or prior purification. Matrix, such as powder or plant material, is dissolved or extracted with common solvents and deposited directly on disposable PaperSpray® cartridges for high-throughput, automated analysis. The combination of PaperSpray® sample introduction and High Resolution Accurate Mass Spectrometry (HRAM) provides a powerful and simple tool for identification of new substances, without requiring reference standards.

## Highlights

• PaperSpray-MS has been applied to the detection and identification of novel psychoactive substance (NPS) in consumer products using minimal sample preparation.
• PaperSpray-MS provides a rapid screening tool and, interfaced to high resolution accurate mass (HRAM), a powerful identification technique for obtaining chemically-relevant information.
• Identification of second and third generation synthetic cannabinoids was accomplished by accurate mass interpretation and interpretation of spectra from data dependent MS^2^ analyses.
• The combination of these techniques provides a simplified work flow for detection and identification of NPS by accurate mass and confirmation by MS^2^ without the necessity of reference standards.
• HRAM also allows the identification of unknown compounds outside the target compound class.
• Analysis times for PS–MS are less than two minutes rather than the greater than 12 min with UPLC used here.
• PaperSpray with HRAM detection provided results which were comparable to those from UPLC-MS.

## 1 Introduction

Forensic drug analysis has many challenges; one of them is staying ahead of synthetic chemists. New, pharmacologically active, compounds are being created and made available to the public in inconspicuous ways. Synthetic cannabinoids and cathinones, two common classes of novel psychoactive substances (NPS), have been detected in many herbal incense products and powdered bath salts [1], [2]. These compounds mimic the effects of tetrahydrocannabinol (THC), but often elude detection by current drug screening techniques that require standards or reference spectra. NPS are typically dissolved and sprayed onto plants or powders to facilitate their use. The majority of NPS are synthetic cannabinoids from the Far East and Southeast Asia [3]. Their structures are readily modified, without loss of physiological activity, resulting in new NPS-type compounds that evade regulation as controlled substances. In the United States, many cannabinoids have been regulated via legislation, and sixteen of the first and second generation cannabinoids are listed as Schedule 1 controlled substances [4], [5], [6], [7]. The emergence of these novel compounds mandates the development of testing approaches that incorporate simplicity, structural selectivity, robustness and qualitative reproducibility, as the most critical attributes; requirements that are addressable using mass spectrometric methods. The use of mass spectrometry (MS) and tandem MS interfaced to Ultra-Performance Liquid Chromatography (UPLC) and ambient ionization techniques, such as Direct Analysis in Real Time (DART), for the identification of these emerging compounds has been reported [8], [9], [10]. The use of High-Resolution Mass Spectrometry (HRMS) for non-targeted analysis of designer drugs has been proposed to keep pace with their continual evolution [11], [12], [13]. HRMS offers enhanced specificity over conventional MS, and improvements in software expedite data mining [14]. Analytical standards are required for confirmation, but when standards are not available, these approaches narrow the list of possible compounds. Several investigators have proposed Gas Chromatography–Tandem Mass Spectrometry (GC–MS/MS) methods as a means for differentiation of isomeric cannabinoids [15], [16]. JWH-250, JWH302, and JWH-201 are all isomeric and difficult to distinguish by GC alone. In these cannabinoids, the methoxy group differs in its position on the aromatic ring versus the indole substituent. However, some common fragments in each cannabinoid were found to have different ratios and, thus, ortho methoxy (JWH-250), meta methoxy (JWH-302) and para methoxy (JWH-201) could be individually distinguished [17]. Ultimately, in an attempt at standardization, a recommended methodology has been provided by the United Nations Office on Drugs and Crime for identification and analysis of synthetic cannabinoid agonists in seized materials [18]. In this report, Thin-Layer Chromatography (TLC) developing systems with *R*_f_ values for cannabinoids, Ion Mobility Spectrometry (IMS) data with cannabinoid drift times, and GC–MS conditions with retention times for selected cannabinoids, are presented. A Liquid Chromatography–Tandem Mass Spectrometry (LC–MS/MS) methodology, with quantitation using internal standards and 5-point calibration curves is detailed, as well as sample preparation techniques for cannabinoids in herbal blends.

An alternative ambient method for ionization and sample introduction into the mass spectrometer is PaperSpray [19], [20]. Initially described in 2010, PaperSpray (PS) uses a cellulosic substrate shaped to a fine tip that produces a spray when solvent and high voltage are applied [21], [22]. Applications of PS–MS include the analysis of dried blood spots for monitoring drugs of abuse, immunosuppressant drugs (e.g. tacrolimus) or cocaine residue on different surfaces [23], [24]. A comprehensive review of PS–MS applications as well as limitations of the technique has been recently published [25]. Although PS–MS is capable of detecting drugs and metabolites directly in biofluids, it is performed without chromatography and, thus, HRMS, MS/MS or a combination of both is necessary for structural confirmation. As with other ambient ionization techniques, selectivity and specificity are heavily dependent on the capabilities of the mass spectrometer. In many cases, direct methods cannot distinguish diastereomers, or closely related structural isomers that fragment in a similar manner, without prior HPLC separation, which prevents labile metabolites such as acyl glucuronides from decomposing in the source region resulting in an inflated concentration of the parent drug. Methodology adjustments to improve the selectivity of PS–MS without significantly increasing the analysis time include the addition of derivatization agents to the paper substrate prior to analysis [26], [27]. Such modifications are intended to derivatize specific functional groups to aid in distinguishing closely related structural isomers. One emerging technique that may improve selectivity for PS–MS analysis is Ion Mobility Spectrometry–Mass Spectrometry (IMS–MS), which separates analytes in the gas phase prior to entering the mass spectrometer. Alternatively, FAIMS–PS–MS/MS was recently reported to have been used for the separation of the structurally similar opiate isomers: morphine, norcodeine and hydrocodone [28].

In this report, we combine PS with High Resolution Accurate Mass (HRAM) and Full Scan-data-dependent MS^2^ (FS-ddMS^2^) as a high throughput screening technique for identification of the NPS in herbal matrices. The sample preparation employed involves dissolution with or without extraction from the matrix. Combining PS ionization with HRAM and FS-ddMS^2^ provides a powerful and potentially high-throughput tool for identification and quantification of synthetic cannabinoids, as well as other drugs, without extensive sample preparation or chromatographic separation. PS is demonstrated to address some of the principal needs of the qualitative screens for NPS in crude sample preparations, including simplicity, availability of structural information, comparable mass spectra to reference standards, and high correlation with LCMS results, but with considerable time savings (i.e. 2 min vs. 12 min).

## 2 Experimental

### 2.1 Materials

Acetonitrile and methanol were HPLC grade and purchased from VWR Scientific. Acetic acid (A.R. grade) was purchased from VWR scientific. Reference standards for XLR-11, UR-144, A-796260, PB-22, AB-PINACA, and MAM-2201 were purchased from Cerilliant Analytical Reference Standards, Round Rock TX. Specimens of herbal incense and powders were acquired from local sources and were stored at ambient temperature. Samples for UPLC-ToF-MS were transferred to a glass tube, a 1:1 mixture of methanol/acetonitrile was added, sonicated 10 min, and then diluted 1:50 with acetonitrile: DI water (20:80). Aliquots were transferred to auto sampler vials for analysis. Extracts were stored under refrigeration for future use. Ten microliters of refrigerated extract were deposited on Velox™ Sample Cartridges (Prosolia, Indianapolis, IN) and allowed to dry under ambient conditions. The PS cartridges were stored with drying agent at room temperature prior to analysis. Samples were prepared in Indianapolis, Indiana and shipped to San Jose, California for analysis.

### 2.2 Instrumentation

#### 2.2.1 PS MS

A Velox 360™ PaperSpray System (Prosolia) was interfaced to a Thermo Scientific™ Q-Exactive™ Focus high-resolution, accurate-mass (HRAM) mass spectrometer (Thermo Fisher Scientific, San Jose, CA) for analysis. A solvent mixture consisting of acetonitrile/water/acetic acid (90/10/0.1 v/v/v) was used as the eluting solvent and spray solvent for the PaperSpray source. Voltages for the mass spectrometer in the PS experiment were set using tune files from the mass spectrometer. The Q-Exactive MS was operated in full-scan data-dependent MS^2^ mode. In confirmation mode, data-dependent MS^2^ scans are triggered based on detection of compounds in an inclusion list. Data were acquired with Thermo Scientific TraceFinder™ software (v 3.2) over a mass range of *m*/*z* 175–500 and resolution of 70,000. During sample analysis, the source voltage was provided by the mass spectrometer and was ramped from 0 kV (0.1 min) to 5 kV (hold for 0.7 min) then back to 0 kV (0.1 min) followed by a negative pulse of −4.5 kV (0.1 min).

#### 2.2.2 UPLC-ToF

Chromatographic separation was completed using a Waters (Milford, MA) Acquity UltraPerformance® Liquid Chromatograph. The UPLC separations were performed using BEH C18 column (2.1 × 100 mm, 1.8 μm particle size) with a gradient elution at a flow rate of 0.5 ml/min. The UPLC mobile phase consisted of DI water containing 0.05% formic acid (solvent A) and acetonitrile (solvent B). The gradient profile was 42% B for 0.3 min, linear increase to 97% B in 11 min, hold for 0.5 min, then reverse to 42%B. Electrospray ionization mass spectrometry was performed using a Waters LCT Premier XE Time of Flight mass spectrometer. Low voltage scan was used for precursor mass identification followed by collision induced dissociation (CID).

## 3 Results and discussion

**Fig. 1:**
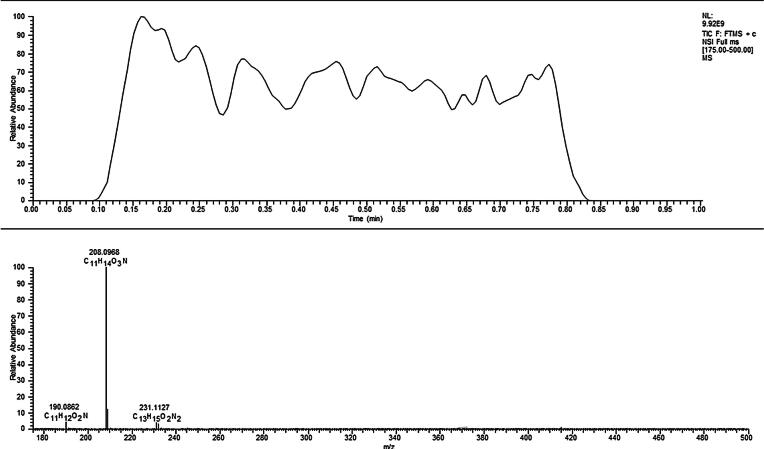
Chronogram from Tranquility (Top) with average HRAM Spectrum for Methylone (bottom).

The herbal samples were analyzed and chronograms were obtained similar to that in Fig. 1 for the Tranquility sample. Integration or summation of the resultant signal across time allowed for extraction of MS or MS/MS spectra. Methylone, the mass spectrum of which is included in Fig. 1, was ultimately identified as a major component in this specimen based on accurate mass. This mass spectrum represents an average over the entire chronogram from 0.10 to 0.83 min. Cannabinoid identification was facilitated by use of an inclusion list and MS^2^ data. Table 1 provides a summary of [*m*/*z*] for suspected cannabinoids as well as [*m*/*z*] values for the detected cannabinoids found in the herbal mixtures. Several suspected cannabinoids were not detected in the herbal mixes. Identification and confirmation of the cannabinoids were based on accurate mass matching (tolerance of 10 ppm) and formula confirmation derived from the HRAM data followed by data dependent fragmentation for any compounds detected on the inclusion list. Two additional compounds, methalone and 5/6 APB, were not contained within in the original inclusion table used for analysis. However, both compounds were tentatively identified post-analysis based on HRAM. Both of these structures were subsequently identified in the herbal blends using UPLC-ToF-MS. As many of these compounds are not in spectral libraries, comparative identification of the compounds was to be inferred from the MS^2^ experimental data. This can be achieved by comparison of the spectra from unknowns with those from knowns and inferring structural homology.

**Table 1:** Inclusion list of compounds.

Theoretical (*m*/*z*) [M+H]^+^	Measured (*m*/*z*) [M+H]^+^	Molecular formula	Compound
176.2270	176.1069	C_11_H_14_ON	5/6 APB⁎
195.1254	–	C_11_H_16_NO_2_	MDMA
208.2258	208.0968	C_11_H_14_O_3_N	Methylone⁎
232.1696	232.1696	C_15_H_21_NO	a-PVP
248.1645	248.1645	C_15_H_21_NO_2_	Methoxetamine
276.1594	–	C_16_H_21_NO_3_	MDPV
312.2322	312.2325	C_21_H_29_NO	UR-144
319.2632	–	C_21_H_34_O_2_	CP 47 497
322.1802	–	C_21_H_23_NO_2_	RCS-4
323.2118	–	C_21_H_26_N_2_O	Acetylfentanyl
328.1696	328.1696	C_23_H_21_NO	JWH-015
328.1696	328.1696	C_23_H_21_NO	JWH-073
329.2111	–	C_21_H_28_O_3_	HU-331
330.2228	330.2226	C_21_H_28_FNO	XLR-11
331.2129	331.2258	C_18_H_26_N_4_O_2_	AB-PINACA
336.1361	336.1360	C_18_H_22_ClNO_3_	25C-NBOMe
336.1958	336.1951	C_22_H_25_NO_2_	JWH-250
339.1703	–	C_20_H_22_N_2_O_3_	URB597
340.1463	340.1908	C_21_H_22_ClNO	JWH-203
342.1852	342.1693	C_24_H_23_NO	JWH-018
345.2285	–	C_19_H_28_N_4_O_2_	ADB-PINACA
348.9738	348.9737	C_15_H_10_BrClN_2_O	Phenazepam
355.2380	355.2380	C_22_H_30_N_2_O_2_	A796,260
356.2009	356.2207	C_25_H_25_NO	JWH-019
356.2009	356.2207	C_25_H_25_NO	JWH-122
357.2285	357.2281	C_20_H_28_N_4_O_2_	AB Chminaca
359.1754	359.1753	C_23_H_22_N_2_O_2_	QUPIC (PB-22)
360.1758	360.1759	C_24_H_22_FNO	AM-2201
369.1721	369.1718	C_20_H_21_FN_4_O_2_	AB-FUBINACA
370.2165	370.2165	C_26_H_27_NO	JWH-210
372.1958	372.3621	C_25_H_25_NO_2_	JWH-081
374.1915	374.2408	C_25_H_24_FNO	MAM-2201
376.2271	376.2250	C_25_H_29_NO_2_	RCS-8
377.1660	377.1656	C_23_H_21_FN_2_O_2_	5F-PB-22
380.0856	–	C_18_H_22_BrNO_3_	25B-NBOMe
383.1878	–	C_21_H_23_FN_4_O_2_	ADB-FUBINACA
385.1911	–	C_25_H_24_N_2_O_2_	JWH-200
387.2894	–	C_25_H_38_O_3_	HU-211
427.2016	–	C_27_H_26_N_2_O_3_	WIN 55-212-2
428.0717	428.0717	C_18_H_22_INO_3_	25I-NBOMe
459.0928	459.0913	C_22_H_23_IN_2_O	AM-2233

⁎ Compounds were not included in original Inclusion List. (–) indicates compound was not detected in blends.

**Fig. 2:**
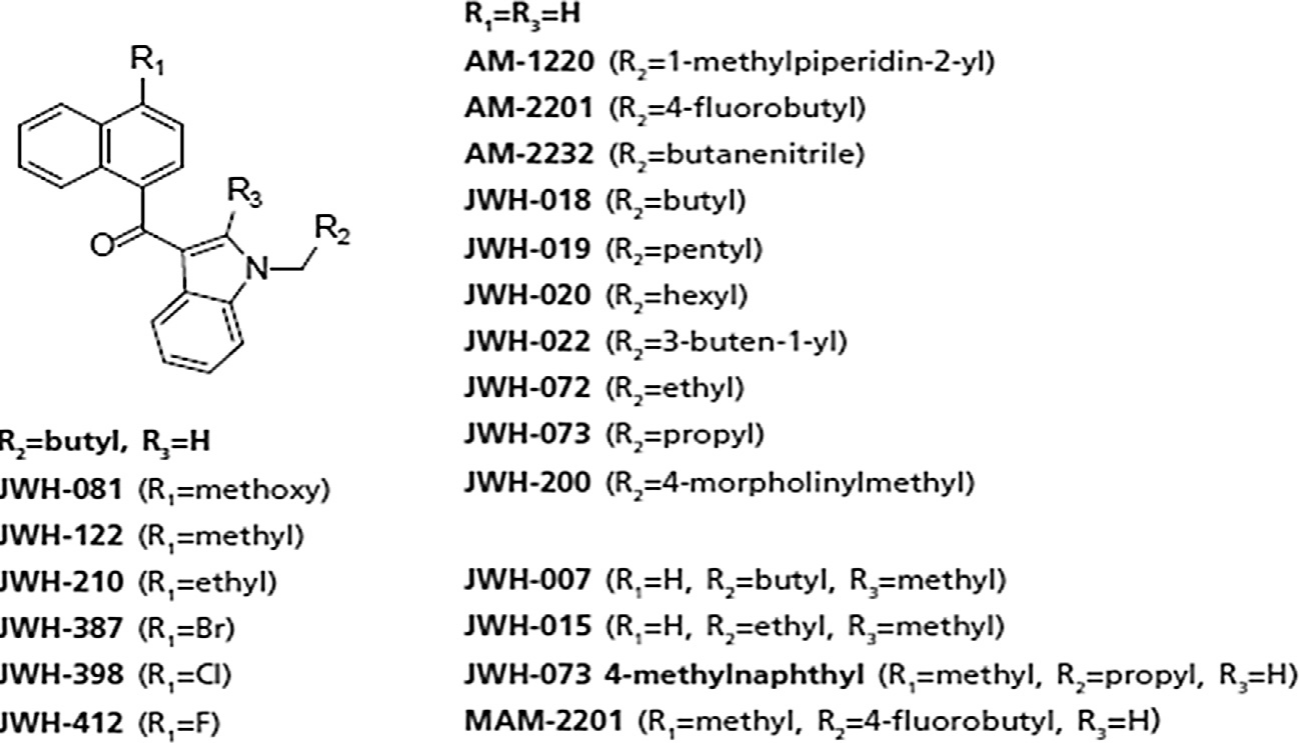
Structures of first and second generation synthetic cannabinoids.

**Fig. 3:**
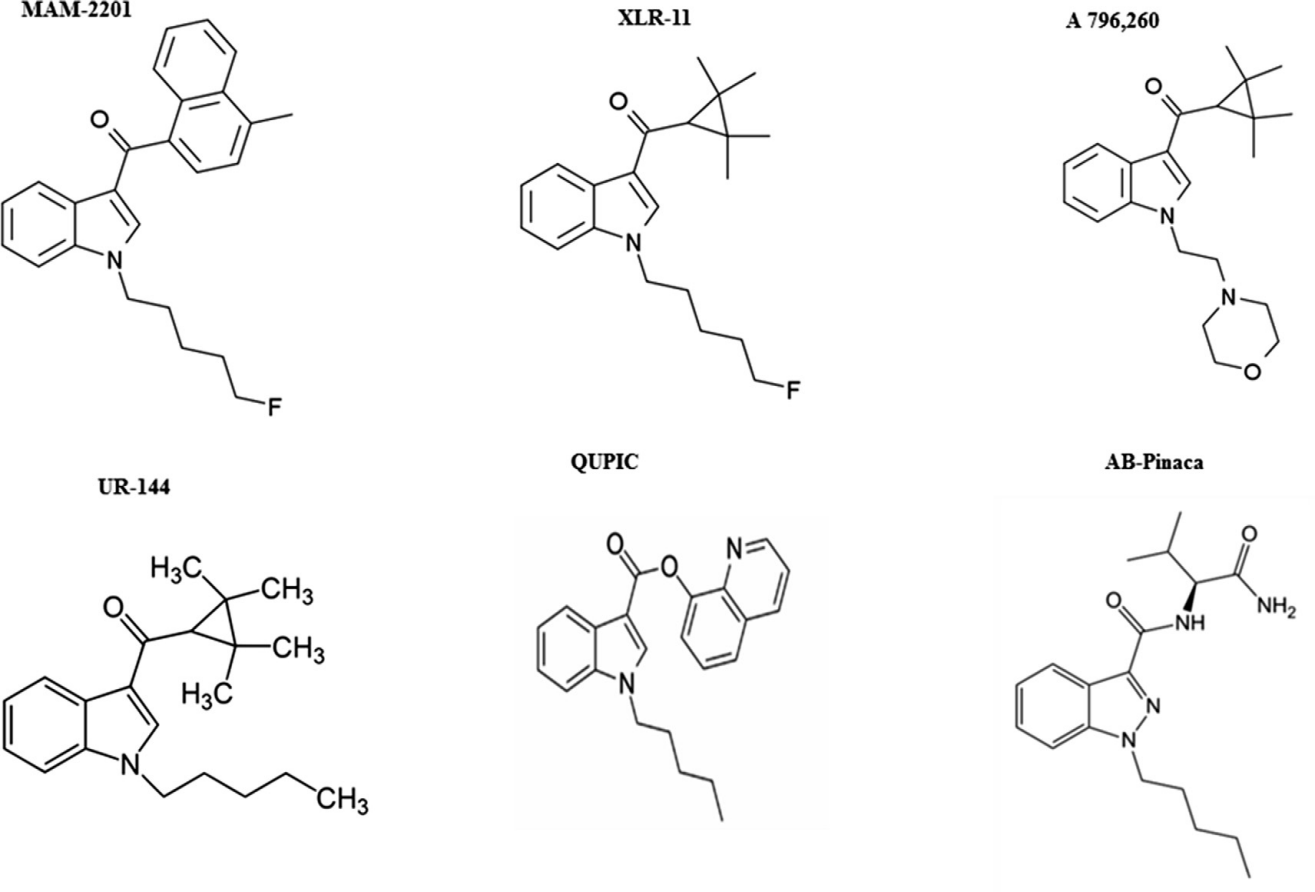
Structures of third generation synthetic cannabinoids.

**Fig. 4:**
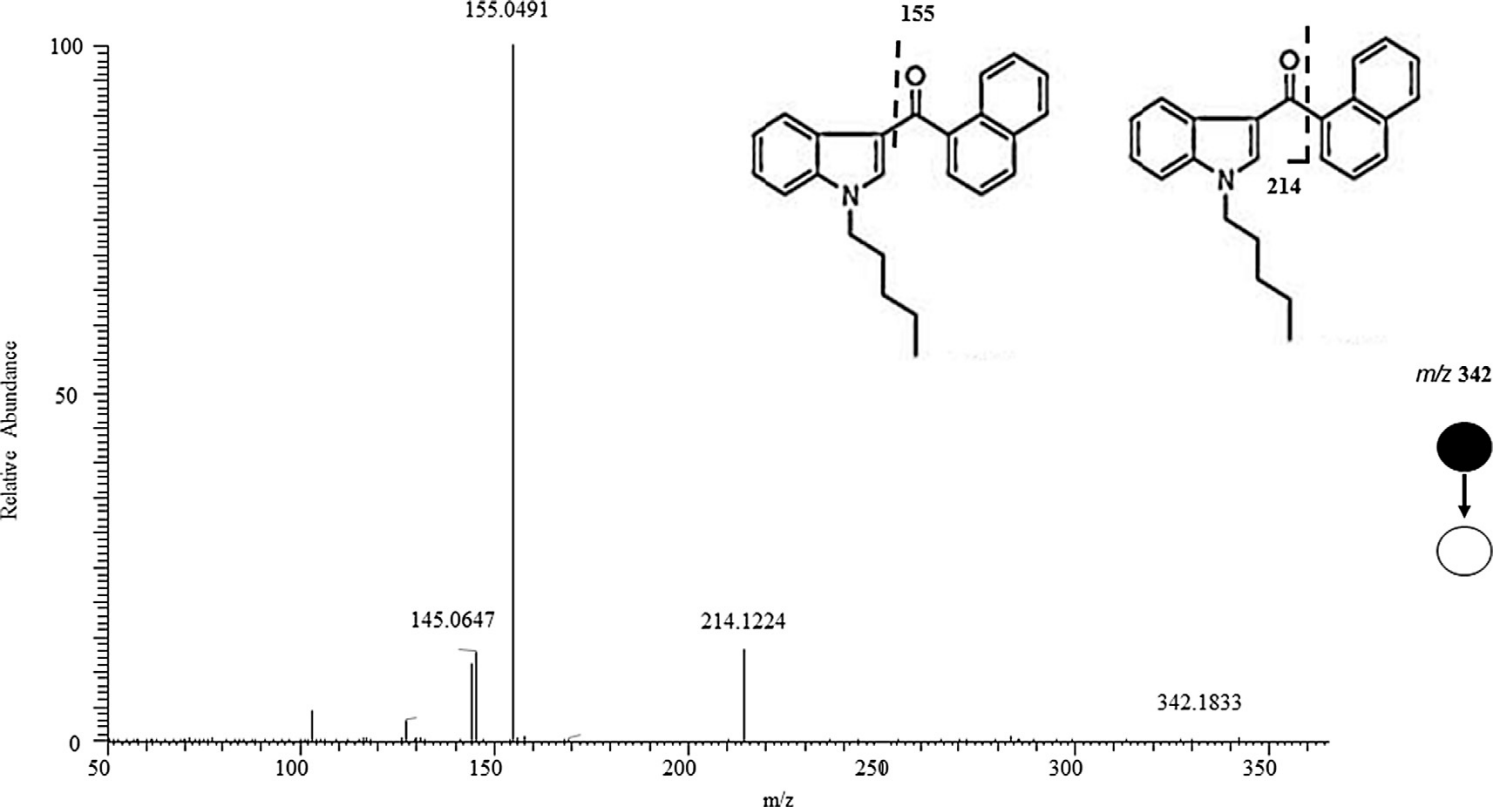
Structure and MS^2^ spectrum from JWH-018.

**Fig. 5:**
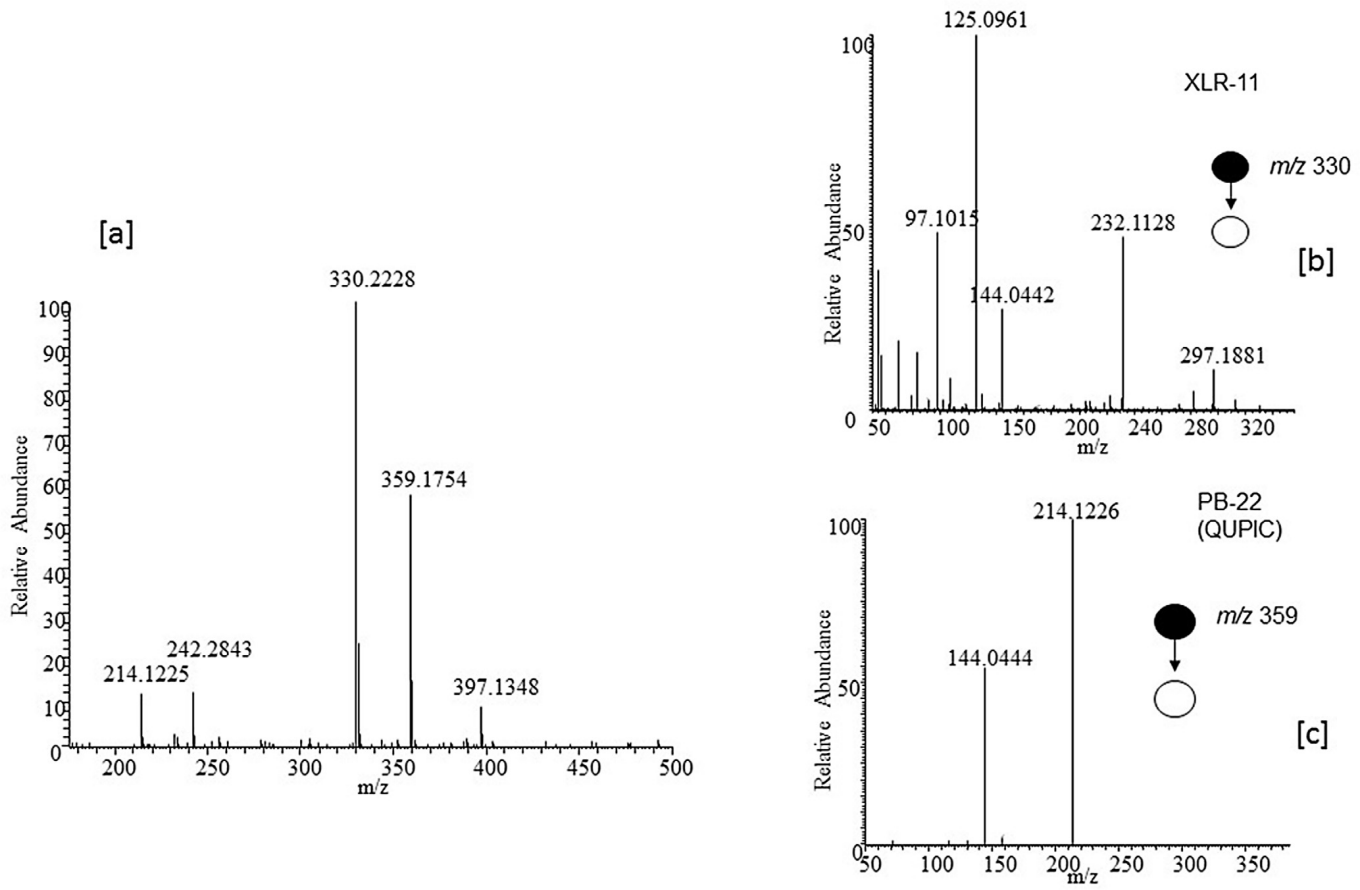
FSddMS^2^ spectra from Bizarro specimen. [a] XLR-11 *m*/*z* 330.2228 C_21_H_29_ONF and PB-22 *m*/*z* 359.1754 C_23_H_33_O_2_N_2,_ [b] MS^2^ for XRL-11, and [c] MS^2^ for PB-22.

**Fig. 6:**
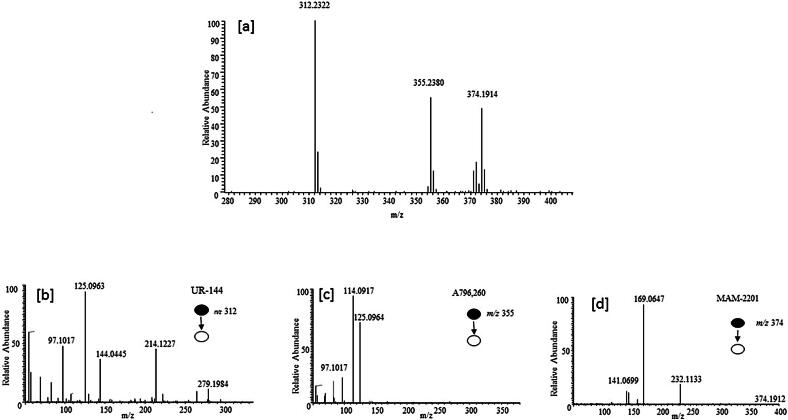
FSddMS^2^ spectra from Black Magic Smoke showing the predictable fragmentation behavior of the cannabinoids. [a] UR-144 *m*/*z* 312.2322 C_21_H_30_ON, A796,260 *m*/*z* 355.2380 C_22_H_31_O_2_N_2_, and MAM 2201 *m*/*z* 374.1914 C_25_H_25_ONF, [b] MS^2^ for UR-144, [c] MS^2^ for A796,260, [d] MS^2^ for MAM 2201.

The structural similarities among the first and second generation cannabinoids [11] (Fig. 2) and the newly emerging third generation [8] (Fig. 3) are apparent and will be leveraged in interpreting spectral information and identifying the compounds detected in the consumer products analyzed here. MAM-2201 (second generation synthetic cannabinoid) is similar to typical naphthoylindole structure observed in earlier cannabinoids with a fluorine added to the alkyl chain. However, A796260, UR-144 and XLR-11 (Fig. 3) contain completely new chemical moieties adjacent to the keto-indole core. In the MS^2^ experiment, the cannabinoids studied here all fragment in a predictable manner allowing MS^2^ data to more readily identify the location and nature of the substituent in the new compound. JWH-018 is one of the most commonly found and well-studied cannabinoids [12]. This allows its MS^2^ behavior (Fig. 4) to facilitate the interpretation of other spectra with fragmentation on either side of the central carbonyl to yield *m*/*z* 155.0491 (C_11_H_7_O) and *m*/*z* 214.1224 (C_14_H_16_NO) being diagnostic of substituents. Consequently, it would be predicted that XLR-11 would produce fragment ions at *m*/*z* 125.0961 (C_8_H_13_O) and *m*/*z* 232.1128 (C_14_H_15_ONF). These ions are, in fact, observed in the MS^2^ spectrum of this compound as illustrated in Fig. 5. The sample containing XLR-11 also contains QUPIC or PB-22. PB-22 is an analog of JWH-018 with an ester at the indole-3 position and 8-hydroxyquinoline replacing the naphthalene group. Fragmentation is similar to JWH-018 with *m*/*z* 214.1226 (C_14_H_16_ON) and 144.0444 (C_9_H_6_ON) being predominant. Mass spectral data from analysis of the Black Magic Smoke specimen is illustrated in Fig. 6. The data from this specimen were consistent with a mix of UR-144, A796,260, and MAM-2201. Specifically, UR-144 fragments are *m*/*z* 125.0963 (C_8_H_13_O) and 214.1227 (C_14_H_16_ON), representing cleavage at the ketone to generate the epoxide containing fragments. The common *m*/*z* 125.0963 and unique *m*/*z* 214.1227 and 232.1128 ions are consistent with the purported structural difference minus F for H substitution between UR-144 and XLR-114. UR-144 does not contain a fluorine as seen in XLR-11 and thus *m*/*z* 214.1227 versus *m*/*z* 232.1128 is present. The primary fragments observed in A796260 were *m*/*z* 114.0917 (C_6_H_12_ON), *m*/*z* 125.0964 (C_8_H_13_O) and also *m*/*z* 257.1278 (C_15_H_17_O_2_N_2_). MAM-2201 fragments were as expected *m*/*z* 169.0647 (C_12_H_9_O) as well as *m*/*z* 232.1133 (C_14_H_15_ONF). Additional confirmation of structure was obtained by comparison of mass spectral data from selected reference standard cannabinoids summarized in Table 2. In all of these compounds, the mass spectrum from extracted samples and reference standards were comparable. Overall, the analysis using PS included 42 samples from post-mortem crime scenes and resulted in the detection of numerous designer cannabinoids including: AM-2201, JWH-210, JWH-250, MAM-2201, RCS-8, A796260, UR-144, JWH-122, JWH-019, AM-2233, XLR11, PB-22.

**Table 2:** Parent and product ions from selected cannabinoid reference standards.

Compound	Molecular formula	Theoretical (*m*/*z*) [M+H]^+^	Observed (*m*/*z*) [M+H]^+^	Fragment 1 (*m*/*z*) [M+H]^+^	Fragment 2 (*m*/*z*) [M+H]^+^
UR-144	C_21_H_29_NO	312.2322	312.2318	214.1226	125.0963
XLR-11	C_21_H_28_FNO	330.2228	330.2222	232.1130	125.0962
AB-PINACA	C_18_H_26_N_4_O_2_	331.2129	331.2121	233.1281	215.1177
A796260	C_22_H_30_N_2_O_2_	355.2380	355.2374	125.0963	114.0917
PB-22	C_23_H_22_N_2_O_2_	359.1754	359.1748	214.1224	144.0442
MAM2201	C_25_H_24_FNO	374.1915	374.1910	232.1131	169.0646

These samples were also analyzed by UPLC-ToF-MS methodology [8]. The total run time per sample was 12 min as compared to <2 min using PS-MS. In-source, collision-induced dissociation of precursor was used in the UPLC-ToF-MS experiments to identify the compounds in the different blends. A summary of results from PS–MS and UPLC-ToF-MS is presented in Table 3. These data show a strong correlation between the two techniques in which NPS were detected and in the identification capabilities of the two techniques. Differences in the number of synthetic cannabinoids in several blends were obvious and were attributed to differences in concentration of analyzed sample or, possibly, sample stability. The UPLC-ToF-MS data were obtained from fresh extracts and diluted (1:50) prior to analysis. The PS–MS analysis was performed on extract solutions that had been refrigerated several months prior to analysis and solutions were not diluted.

**Table 3:** Summary of NPS detected by UPLC-ToF-MS and PS–MS data.

Specimen	UPLC-ToF-MS	PS–MS
Purple Diesel	MAM-2201	MAM-2201
Diablo	MAM-2201	MAM-2201
Funky Green Stuff	UR-144	JWH-081, MAM-2201, A796,260, UR-144
Assassin	MAM-2201, UR-144	UR-144, MAM-2201
Black Magic Smoke	UR-144	MAM-2201, A796,260, UR-144
Darkness Blueberry	UR-144	MAM-2201, A796,260, UR-144
Gorilla Pro GD	JWH-081, JWH-210, MAM-2201, UR-144	MAM-2201, A796,260, UR-144
Black Rooster	AM-2201	MAM-2201, AM-2201
Funky Monkey	AM-2201, JWH-210, JWH-250	AM-2201, JWH-210, JWH-250, MAM-2201, RCS-8
Matrix	JWH-122	JWH-122
Bayou Blaster	AM-2201, AM-2233, JWH-210	AM-2201, AM-2233, JWH-210
K2 XXX Chronic	JWH-122, JWH-203	JWH-122, JWH-203
Cloud 9	AM-2201, JWH-019, JWH-122, JWH-250	AM-2201, JWH-019, JWH-122, JWH-250
Demon	5F-PB-22, PB-22	PB-22, AM2201
Colorado	AM-2201	AM2201, XLR11, A796,260
iBlown 4G	XLR11, XLR11 N-4-pentenyl derivative	XLR11, A796,260, AM-2201
Sunshine Daydream	UR-144, XLR11	UR-144, XLR11
Joker	5F-PB-22, PB-22	PB-22
Sunshine Nightmare	UR-144, XLR11	UR-144, XLR11, PB-22
Ultra-Zombie Matter	AM-2201, JWH-210, Phenazepam	JWH-210
Crazy Monkey	AM2201	AM-2201
No Mames	AB-PINACA	AB-PINACA, AM-2201
Brain Freeze	AB-FUBINACA, AB-PINACA	AB-PINACA
Unspecified Blotter Paper	25C-NBOMe, 25H-NBOMe, 25I-NBOMe	25C-NBOMe, 25I-NBOMe
Crystal Clean Hookah Cleaner	Alpha-PVP	Alpha-PVP
Bliss Ultra	Methoxetamine	Methoxetamine
Super Flame	5F-PB-22, FUB-PB-22, PB-22	5F-PB-22, FUB-PB-22, PB-22
Inferno	5F-PB-22, PB-22	5F-PB-22, PB-22
Bizarro	XLR11	XLR11, PB-22
Black Diamond	5F-PB-22, AB-FUBINACA, AB-PINACA, PB-22, XLR11	5F-PB-22, AB-FUBINACA, AB-PINACA, PB-22, XLR11
E-cigarette Liquid 2	5F-PB-22, AB-CHMINACA, PB-22, XLR11	AB-CHMINACA, PB-22
OMG	XLR11	XLR11
WTF	XLR11	XLR11
Scooby Snax	None Detected	None Detected
Mr. Nice Guy	AM-2201, JWH-018, JWH-081, JWH-210, MAM-2201	AM-2201, JWH-018, JWH-210, MAM-2201
Caution Yellow	UR-144, XLR11	UR-144, XLR11
Mind Candy	5/6-APB	5/6-APB
Speed Rush	Alpha-PVP	Alpha-PVP
Tranquility	Methylone	Methylone
K2 Blonde	–	JWH-018. JWH-073
White Dragon	–	JWH-018
Spike Silver	–	JWH-018

## 4 Conclusions

PS–MS has been applied to the detection and identification of NPS compounds in consumer products using minimal sample preparation. PS–MS interfaced to HRAM provides a rapid screening tool and a useful technique for obtaining chemically-relevant structural information without chromatographic separation. Chromatography is necessary for conformation of isobaric compounds or other structural isomers, although this is a limitation for all ambient ionization techniques, as these types of compounds cannot be distinguished by mass spectrometry alone. Future combinations of IMS–MS with PS ionization may eliminate the need for prior separation of isobars. Identification of the synthetic cannabinoids was accomplished by accurate mass interpretation and inference and interpretation of spectra from data dependent MS^2^ analyses. The combination of these techniques provides a simplified work flow for detection and identification of NPS by accurate mass and MS^2^ fragmentation when reference standards are not readily available. The full scan and MS^2^ spectrum from selected cannabinoids were compared to reference standards for additional confirmation. HRAM also allows the identification of unknown compounds outside the target compound class, as in the case of methylone in the Tranquility sample and 5/6 APB in Mind Candy samples (both confirmed by UPLC-ToF-MS). Methylone and its isomers could not be distinguished individually using PS–MS. However, isomeric forms were not detected in the UPLC-ToF-MS analysis and, as of April 12, 2013, all forms of methylone were listed as Schedule 1 in the Federal register. These data demonstrate the possibility of the combined techniques for targeted, as well as non-targeted, analysis. Analysis times for PS–MS are <2 min, rather than the >12 min with UPLC used here. PS–MS with HRAM detection provided results that were comparable to those obtained from UPLC-ToF-MS, but with a shorter analysis time and without chromatographic separation.

## Conflict of interest

The authors wish to confirm that there are no known conflicts of interest associated with this publication and there has been no significant financial support for this work that could have influenced its outcome.
